# Deletion of Smad3 protects against diabetic myocardiopathy in db/db mice

**DOI:** 10.1111/jcmm.16464

**Published:** 2021-03-17

**Authors:** Li Dong, Jian‐Chun Li, Zhong‐Jing Hu, Xiao‐Ru Huang, Li Wang, Hong‐Lian Wang, Ronald C. W. Ma, Hui‐Yao Lan, Si‐Jin Yang

**Affiliations:** ^1^ Department of Cardiovascular Medicine Research Center of Integrated Traditional Chinese and Western Medicine The TCM Affiliated Hospital of Southwest Medical University Luzhou China; ^2^ Department of Medicine and Therapeutics Li Ka Shing Institute of Health Sciences Lui Che Woo Institute of Innovative Medicine The Chinese University of Hong Kong Hong Kong China; ^3^ Guangdong‐Hong Kong Joint Laboratory on Immunological and Genetic Kidney Diseases Guangdong Provincial People’s Hospital Guangdong Academy of Medical Sciences Guangzhou China

**Keywords:** diabetic myocardiopathy, fibrosis, inflammation, miR‐21, miR‐29, Smad3

## Abstract

Diabetic cardiomyopathy (DCM) is a common diabetic complication characterized by diastolic relaxation abnormalities, myocardial fibrosis and chronic heart failure. Although TGF‐β/Smad3 signalling has been shown to play a critical role in chronic heart disease, the role and mechanisms of Smad3 in DCM remain unclear. We reported here the potential role of Smad3 in the development of DCM by genetically deleting the Smad3 gene from db/db mice. At the age of 32 weeks, Smad3WT‐db/db mice developed moderate to severe DCM as demonstrated by a marked increase in the left ventricular (LV) mass, a significant fall in the LV ejection fraction (EF) and LV fractional shortening (FS), and progressive myocardial fibrosis and inflammation. In contrast, db/db mice lacking Smad3 (Smad3KO‐db/db) were protected against the development of DCM with normal cardiac function and undetectable myocardial inflammation and fibrosis. Interestingly, db/db mice with deleting one copy of Smad3 (Smad3 ± db/db) did not show any cardioprotective effects. Mechanistically, we found that deletion of Smad3 from db/db mice largely protected cardiac Smad7 from Smurf2‐mediated ubiquitin proteasome degradation, thereby inducing IBα to suppress NF‐kB‐driven cardiac inflammation. In addition, deletion of Smad3 also altered Smad3‐dependent miRNAs by up‐regulating cardiac miR‐29b while suppressing miR‐21 to exhibit the cardioprotective effect on Smad3KO‐db/db mice. In conclusion, results from this study reveal that Smad3 is a key mediator in the pathogenesis of DCM. Targeting Smad3 may be a novel therapy for DCM.

## 1 BACKGROUND

Diabetic cardiomyopathy (DCM) has become a leading complication of diabetes mellitus, which eventually results in heart failure (HF) even in the absence of coronary and hypertensive heart diseases.^1^, ^2^ Like most chronic heart diseases, a typical hallmark of diabetic myocardiopathy is left ventricular (LV) dysfunction including increased LV wall thickness and LV mass.^3^ It is well established that the pathological features of DCM are mainly characterized by inflammation and fibrosis. Cardiac inflammation caused by hyperglycaemia and metabolic stress molecules including angiotensin II (Ang II), advanced glycation end products (AGE) and reactive oxygen species (ROS) eventually progresses to myocardial fibrosis.^4^, ^5^ However, the pathologic mechanisms associated with DCM remain poorly understood.

A growing evidence suggests that TGF‐β/Smad3 signalling is a key pathway associated with cardiovascular diseases. TGF‐β1 exerts its biological effects through its receptor‐mediated activation of Smad2 and Smad3, which are negatively regulated by its downstream inhibitory Smad7. In cardiovascular diseases, Smad3 is pathogenic as deletion of Smad3 protects against cardiovascular inflammation and fibrosis under various pathological conditions such as hypertension, myocardial infarction and obese diabetes.^6^, ^7^, ^8^, ^9^, ^10^ In contrast, Smad7 is cardiac protective because loss of Smad7 enhances Ang II‐induced, Smad3‐mediated cardiovascular fibrosis and NF‐κB‐driven inflammation, which is reversed by overexpressing cardiac Smad7.^11^, ^12^ Increasing evidence suggests that Smad7 is an integrated inhibitor to either inhibit Smad3‐mediated cardiovascular fibrosis via its negative feedback loop or to block NF‐kB‐driven inflammation by inducing expression of IkBα, an inhibitor of NF‐kB.^11^, ^12^, ^13^ Under diabetic and hypertensive conditions, many metabolic stress molecules including Ang II, AGE and C‐reactive protein (CRP) can activate Smad3 to mediate fibrosis via both TGF‐β‐dependent and TGF‐β‐independent mechanisms.^14^, ^15^, ^16^ Thus, Smad3 may be essential in the pathogenesis of diabetic and hypertensive complications, which is validated by our recent finding that diabetic db/db mice null for Smad3 are resistant to the development of diabetic nephropathy.^17^ Nevertheless, the pathologic role of Smad3 in DCM remains to be elucidated. Therefore, in the present study, we aimed to define the role and mechanisms of Smad3 in DCM in a mouse model of type‐2 diabetes in which Smad3 gene is deleted from db/db mice.

## 2 MATERIAL AND METHODS

### 2.1 Generation of Smad3 KO‐db/db mice and their littermates

The genotypes and phenotypes of Smad3 KO mice are described previously.^18^ Because both Smad3 null and db/db mice are infertile, to delete the Smad3 gene from db/db mice, we crossbred Smad3^+/−^ mouse (C57BL/6J) with Lepr^+/−^ (db/m) mouse (C57BL/6J) to obtain double‐heterozygous (Smad3^+/−^ db/m) mice and then to generate Smad3WT‐db/m, Smad3KO‐db/m, Smad3WT‐ db/db, Smad3^+/−^ db/db and Smad3 KO‐db/db offspring. The mouse genotypes were determined by PCR using primers specific to Smad3 (Sense, 5′‐CCACTTCATTGCCATATGCCCTG‐3′ and antisense, 5′‐CCAGACTGCCTTGGGAAAAGC‐3′) and db/db (Sense, 5′‐AGAACGGACACTCTTTGAAGTCTC‐3′ and antisense, 5′‐CATTCAAACCATAGTTTAGGTTTGTGT‐3′). Genotyping from each mouse tail DNA was digitally performed in a 96‐well plate with the QIAxcel Advanced System (QIAGEN, Germany), and each mouse phenotype was identified by the corresponding bands detected by the specific Smad3 and db/db primers. All the mice were housed under standard conditions with circadian light/dark cycles and standard feeding. Eight mice from each of the five genotypes were killed at 32 weeks of age for studying. All animal handling and experimental procedures were approved by Institutional Animal Care and Use Committee at the Chinese University of Hong Kong (Ref No.13‐057‐MIS & 17‐178‐MIS).

### 2.2 Blood pressure and cardiac function analysis

Blood pressure was measured using the tail‐cuff method in conscious mice according to the manufacturer's instruction (CODA non‐invasive blood pressure system; Kent Scientific) as described previously.^6^, ^11^, ^12^ Cardiac structure and function were examined by echocardiography by employing a M‐mode Vevo770 ultrasound imaging system (VisualSonics). The LV ejection fraction (LVEF), LV fractional shortening (LVFS) and LV mass were measured as previously described.^6^, ^11^, ^12^

### 2.3 Histology and immunohistochemical staining

The left mouse heart tissues were cut, fixed at 4°C with the methyl Carnoy's solution and paraffin‐embedded tissue sections at 4 µm were evaluated by using haematoxylin and eosin (HE) and Masson's trichrome staining, respectively. For immunohistochemistry, microwave‐based antigen retrieval technique was used as previously described.^6^, ^11^, ^12^ After microwaving, the sections were incubated with primary antibodies against tumour necrosis factor α (TNFα), interleukin‐1β (IL‐1β), MCP‐1, and TGF‐β1 (Santa Cruz Biotechnology), collagen I and collagen III (Southern Biotech), α‐SMA (Sigma), p‐Smad3 (600‐401‐919, Rockland) and F4/80 (Serotec Ltd) for overnight at 4°C. After being washed, the sections were incubated with the corresponding secondary antibodies for one hour at room temperature and signals were visualized with diaminobenzidine and counterstained with haematoxylin. Quantitative analysis of p‐Smad3 or F4/80−positive cells was counted at×40 power field under 0.0625 mm,^2^ and the positive counts were expressed as cells per millimetres squared. In addition, the quantitative image analysis system (Image‐Pro Plus 6.5, Media Cybernetics, Silver Spring, MD) was used to analyse the per cent expression of TNFα, IL‐1β, MCP‐1 and TGF‐β, and deposition of collagen I, III and α‐SMA as previously described.^6^, ^11^, ^12^

### 2.4 Western blot analysis

Total proteins from the left ventricle was isolated using RIPA lysis buffer, and Western blot was conducted as described before.^6^, ^11^, ^12^ Briefly, the membranes were blocked with 3% BSA, followed by incubation at 4°C for overnight with indicated primary antibodies including phospho‐NF‐κB/p65 (ser276), phospho‐IκBα (ser32), IκBα, phospho‐Smad3 (Cell Signaling Technology), Smad3, Smad7, Smurf2, NF‐κB/p65, β‐actin (Santa Cruz Biotechnology), collagen I and III (Southern Biotech), and α‐SMA (Sigma). Subsequently, the membranes were incubated with IRDye 800‐conjugated secondary antibody (Rockland Immunochemicals) and photographed with Odyssey infrared image system (LI‐COR Biosciences). Finally, the intensity of the bands was calculated by the Image J software and normalized against GAPDH.^6^, ^11^, ^12^

### 2.5 Real‐time PCR

Total RNA from heart was extracted by using Trizol Reagent (Invitrogen) according to the manufacturer's recommendations. The primer sequences for mRNAs including TGF‐β1, collagen I, collagen III, IL‐1β, TNFα, GAPDH, U6 and microRNAs such as miR‐21 and miR‐29b used in this study were described previously.^6^, ^11^, ^12^ Real‐time PCR was conducted on the CFX96 PCR System (Bio‐Rad) using SYBR Green. The targeted gene expression was normalized against the intern reference gene GAPDH or U6.

### 2.6 Statistical analysis

Data from these studies were presented as mean ± standard error of mean (SEM), and statistical analyses were performed with SPSS 21.0 (SPSS Incorporation) with the one‐way analysis of variance (ANOVA) and the Newman‐Keuls multiple comparison tests.

## 3 RESULTS

### 3.1 db/db mice deficient for Smad3 are protected against cardiac dysfunction

**FIGURE 1:**
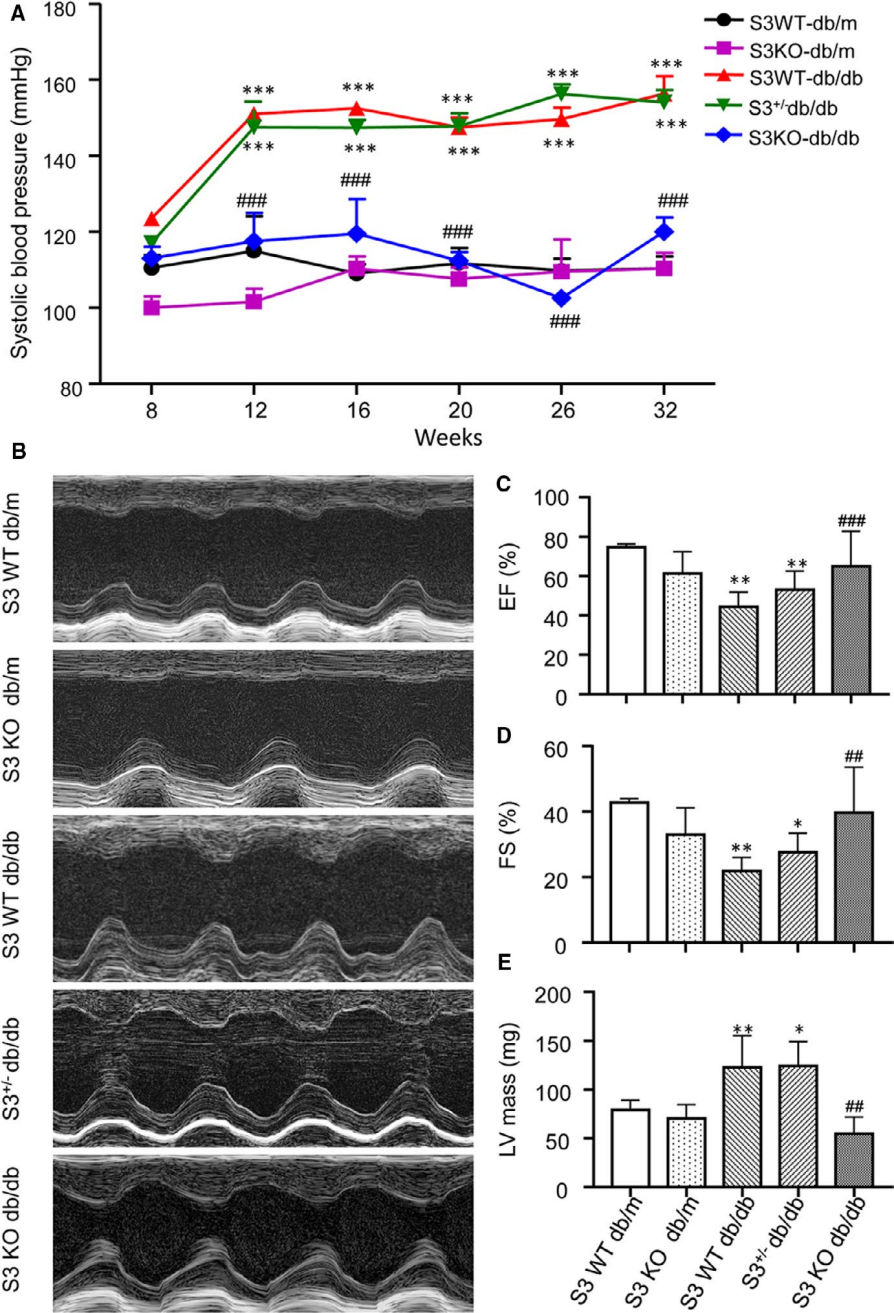
Deletion of Smad3 protects against hypertension and cardiac dysfunction in db/db mice. A, Systolic blood pressure. B, Representative echocardiographic images (M‐mode) of LV function in different phenotypes of db/db or db/m mice. Echocardiographic assessment of the LV ejection fraction (EF; C), LV fractional shortening (FS; D) and LV Mass (E). Values are expressed as mean ± SE for group of eight mice. *P <.05, **P <.01 and ***P <.001 compared with mice at 8 weeks or Smad3(S3) WT‐db/m mice; ^##^ *P* <.01 and ^###^ *P* <.001 compared with Smad3 WT‐db/db and Smad3^+/−^db/db mice

The genotypes of Smad3 KO/WT‐db/db (or db/m) mice and Smad3^+/−^ db/db mice were determined by PCR using genomic DNA as shown in Figure S1. We have previously reported that deletion of Smad3 from db/db mice (Smad3 KO‐db/db) completely protects against the development of the type‐2 diabetic phenotype including the obesity, hyperglycaemia, hyperlipidaemia, and importantly, the glucose intolerance and insulin resistance by largely improving islet β‐cell functions.^19^ Interestingly, we also find that there are no protective effects on the development of type‐2 diabetic phenotypes by disrupting only one copy of Smad3 gene from the db/db mice (Smad3^+/−^db/db), demonstrating an essential role for Smad3 in the pathogenesis of type‐2 diabetes.^19^ Furthermore, we also detected that Smad3 KO‐db/db mice, but not Smad3 WT‐db/db and Smad3^+/−^ db/db mice, were protected from the development of hypertension by maintaining a normal range of blood pressure (Figure 1A). Echocardiography showed that compared to Smad3 WT‐db/m mice, Smad3 WT‐db/db mice exhibited a progressive cardiac dysfunction with a significant decrease in the LVEF and LVFS and a marked increase in the LV mass (all *P* <.001Figure 1A‐E). However, db/db mice with Smad3 deficiency (Smad3 KO‐db/db) preserved a normal cardiac function comparable to the Smad3 KO/WT‐db/m mice (Figure 1 A‐E). Similarly, deletion of one copy Smad3 in Smad3 haploinsufficiency mice (Smad3^+/−^ db/db) failed to show any improvement on cardiac dysfunction (Figure 1 A‐E).

### 3.2 Smad3 deficiency inhibits myocardial fibrosis in db/db mice

**FIGURE 2:**
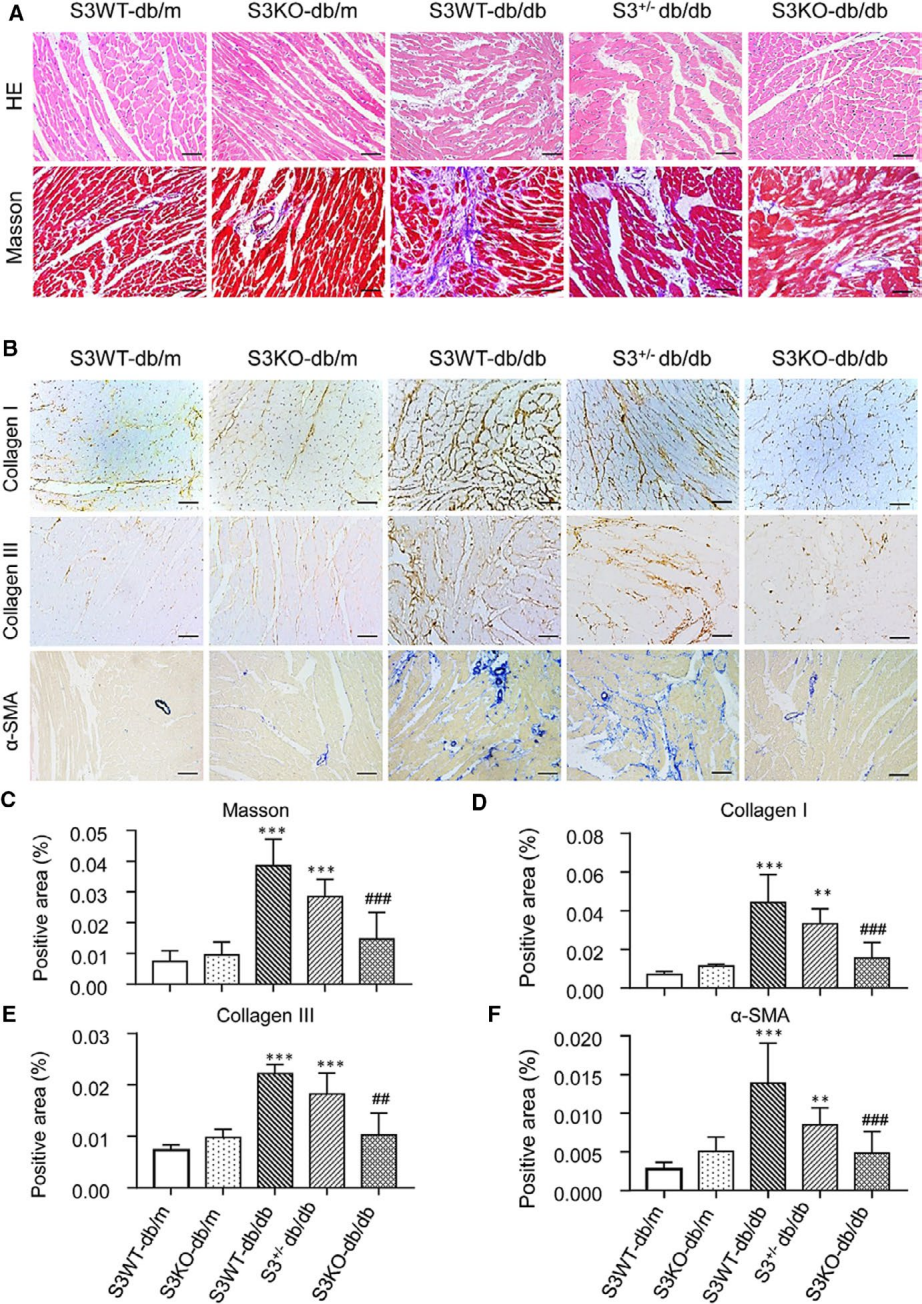
Smad3 deficiency prevents db/db mice from cardiac fibrosis. A, Representative images of H&E and Masson's trichrome staining. B, Representative immunohistochemical images of collagen I, collagen III and α‐SMA. C, Quantitative analysis of Mason's trichrome staining. D‐F, Quantitative analysis of collagen I, collagen III and α‐SMA immunohistochemical staining. Values are expressed as mean ± SE for group of eight mice. **P <.01 and ***P <.001 compared with Smad3(S3) WT‐db/m group; ^##^ *P* <.01 and ^###^ *P* <.001 compared with Smad3 WT‐db/db and Smad3^+/−^db/db groups. Scale bar = 20 µm

**FIGURE 3:**
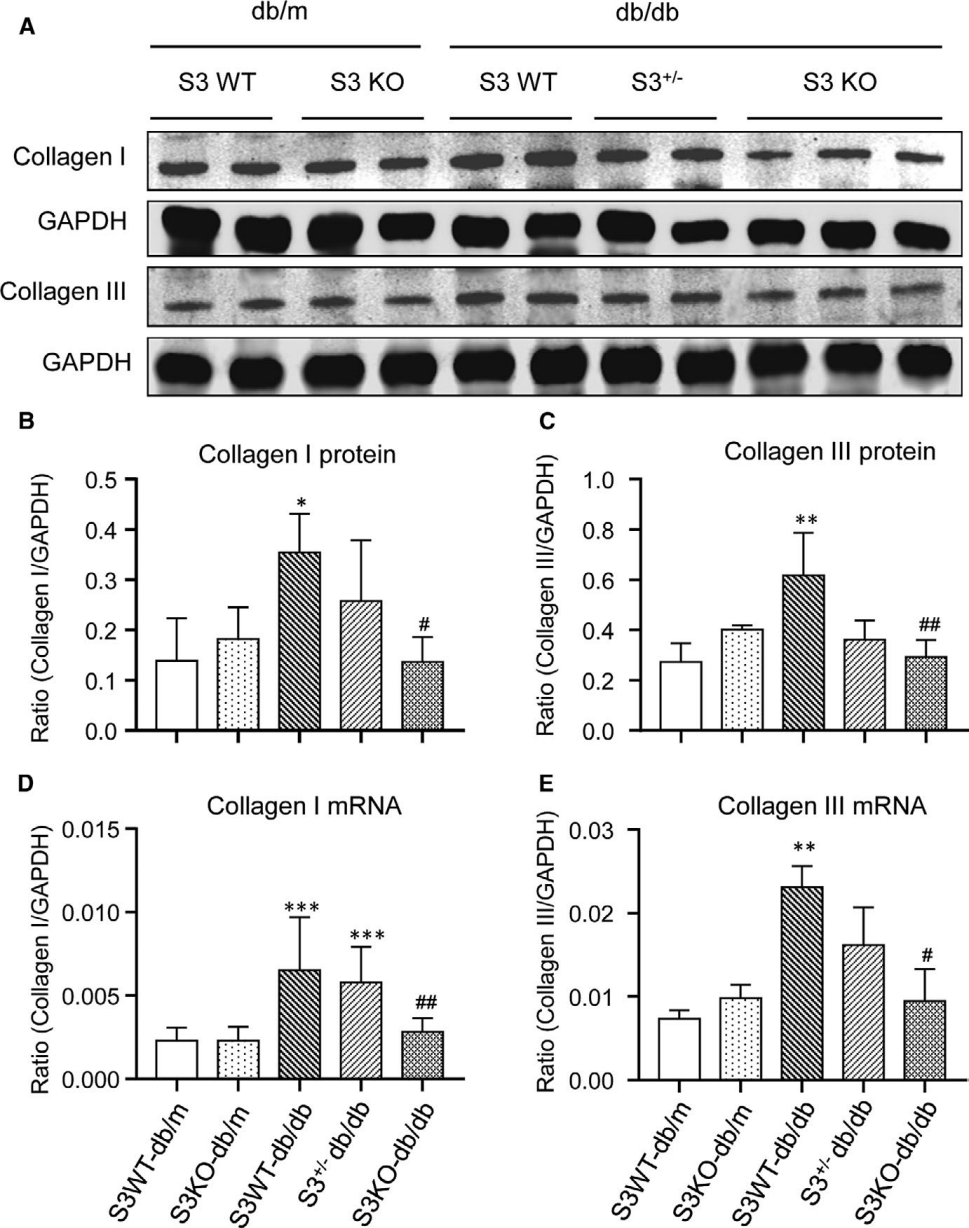
Smad3 deficiency inhibits cardiac collagen I and collagen III accumulation in db/db mice. A‐C, Western blot and quantitative analysis of collagen I and collagen III. D, E, Real‐time PCR analysis of collagen I and collagen III mRNA expression. Values are expressed as mean ± SE for group of eight mice. *P <.05, **P <.01 and ***P <.001 compared with Smad3 (S3) WT‐db/m group; ^#^ *P* <.05 and ^##^ *P* <.01 compared with Smad3 (S3) WT‐db/db and Smad3^+/−^db/db groups

We further determined the protective role of Smad3 deficiency in diabetic myocardiopathy by examining myocardial fibrosis, an indicator of chronic heart disease. Histologically, haematoxylin and eosin (H&E) and Masson's trichrome staining detected that a moderate to severe myocardial fibrosis was developed in Smad3 WT‐db/db mice as well as Smad3^+/−^ db/db mice, which was blunted in db/db lacking Smad3 (Smad3 KO‐db/db Figure 2A,C). Immunohistochemical staining also revealed that a moderate to severe collagen I, collagen III and α‐SMA^+^ myofibroblast deposition in the LV tissues of Smad3 WT‐db/db and Smad3^+/−^ db/db mice were abrogated in Smad3 KO‐db/db mice (Figure 2B,D‐F). Further examinations by Western blot and real‐time PCR also confirmed this notion that a marked elevation in collagen I and collagen III in both transcript and protein levels in Smad3 WT‐db/db and Smad3^+/−^db/db mice was abrogated in Smad3 KO‐db/db mice (Figure 3).

### 3.3 Smad3 deficiency protects against cardiac inflammation in db/db mice

**FIGURE 4:**
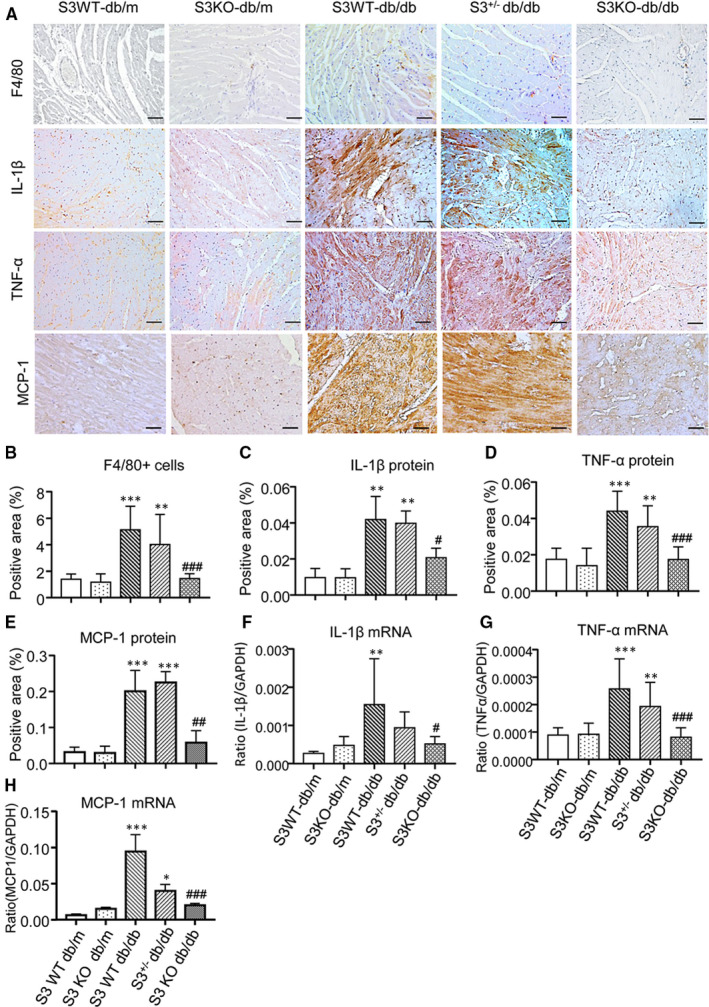
Smad3 deficiency suppresses cardiac inflammation in db/db mice. A, Representative immunohistochemical images of F4/80, IL‐1β, TNF‐α and MCP‐1. B‐E, Quantitative analysis of immunohistochemical staining of F4/80, IL‐1β and TNFα. F‐H, Real‐time PCR analysis of IL‐1β, TNF‐α and MCP‐1 mRNA expression. Values are expressed as mean ± SE for group of eight mice. *P <.05, **P 01 and ***P 001 compared with Smad3 (S3) WT‐db/m group; ^#^ *P* 05, ^##^ *P* 01 and ^###^ *P* 001 compared with Smad3 (S3) WT‐db/db and Smad3^+/−^db/db groups. Scale bar = 20 µm

We further investigated whether deletion of Smad3 inhibits cardiac inflammation in db/db mice. Immunohistochemistry showed that compared to normal control db/m mice, F4/80^+^ macrophage infiltration and expression of IL‐1β, TNFα and MCP‐1 were largely increased in LV tissues of Smad3 WT‐db/db and Smad3^+/−^ db/db mice but were markedly suppressed in Smad3 KO‐db/db mice (Figure 4A‐E). Real‐time PCR also revealed that deletion of Smad3 largely suppressed a marked up‐regulation of IL‐1β, TNFα and MCP‐1 mRNA in Smad3 WT‐db/db and Smad3^+/−^db/db mice (Figure 4F‐H). Collectively, these results clearly demonstrated that Smad3 deficiency protects against the development of cardiac inflammation in db/db mice.

### 3.4 Smad3 deficiency inactivates both TGF‐β/Smad3 and NF‐κB signalling and alters the Smad3‐dependent miRNAs related to myocardial fibrosis

**FIGURE 5:**
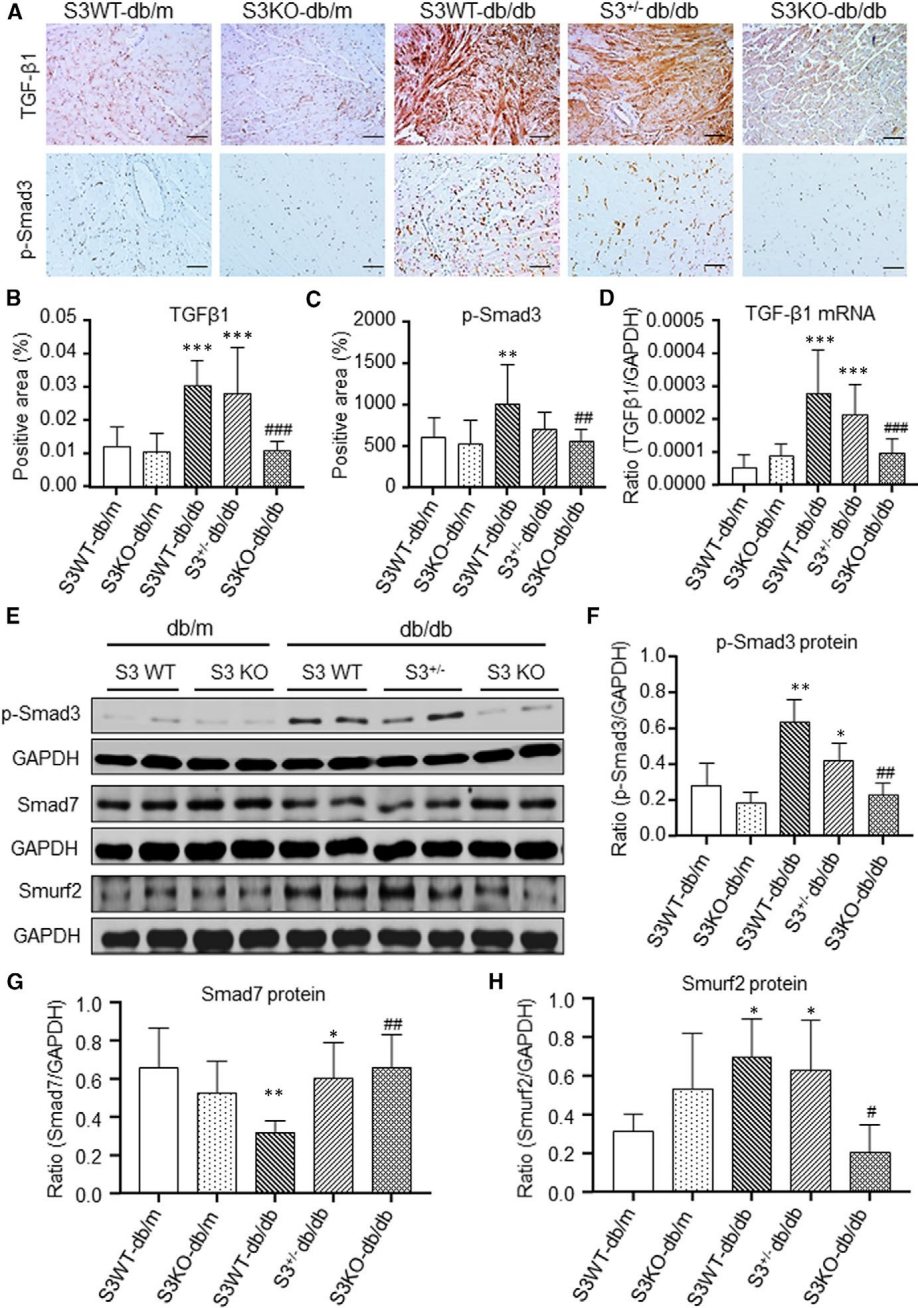
Smad3 deficiency blocks activation of myocardial TGF‐β/Smad3 signalling in db/db mice. A, Representative immunohistochemical images of TGF‐β1 and p‐Smad3. B, C Quantitative analysis of TGF‐β1 and p‐Smad3. D, Real‐time PCR analysis of TGF‐β1 mRNA expression. E‐H, Western blot and quantitative analysis of p‐Smad3, Smad7 and smurf2. Data are presented as mean ± SE for group of eight mice. *P 05, **P 01, ***P 001 compared with Smad3(S3) WT‐db/m group; ^#^ *P* 05, ^##^ *P* 01, ^###^ *P* 001 compared with Smad3 (S3) WT‐db/db and Smad3^+/−^db/db groups. Scale bar = 20 µm

We then studied the potential regulatory mechanisms by which Smad3 deficiency protects against cardiac fibrosis and inflammation in db/db mice. We first determined activation of TGF‐β/Smad3 signalling as it is a key pathway leading to chronic heart failure with progressive myocardial fibrosis.^6^, ^7^, ^8^, ^9^, ^10^ By immunohistochemistry and real‐time PCR, we found that a dramatic increase of TGF‐β1 in LV tissues of Smad3 WT‐db/db and Smad3^+/−^db/db mice resulted in a strong activation of Smad3 as demonstrated by abundant phospho‐Smad3‐nucleated translocation (Figure 5A‐D). In contrast, db/db mice lacking Smad3 showed no up‐regulation of cardiac TGF‐β1 and no activation of TGF‐β/Smad signalling in cardiac tissues (Figure 5A‐D). These observations were further validated by Western blot analysis as shown in Figure 5E,F.

**FIGURE 6:**
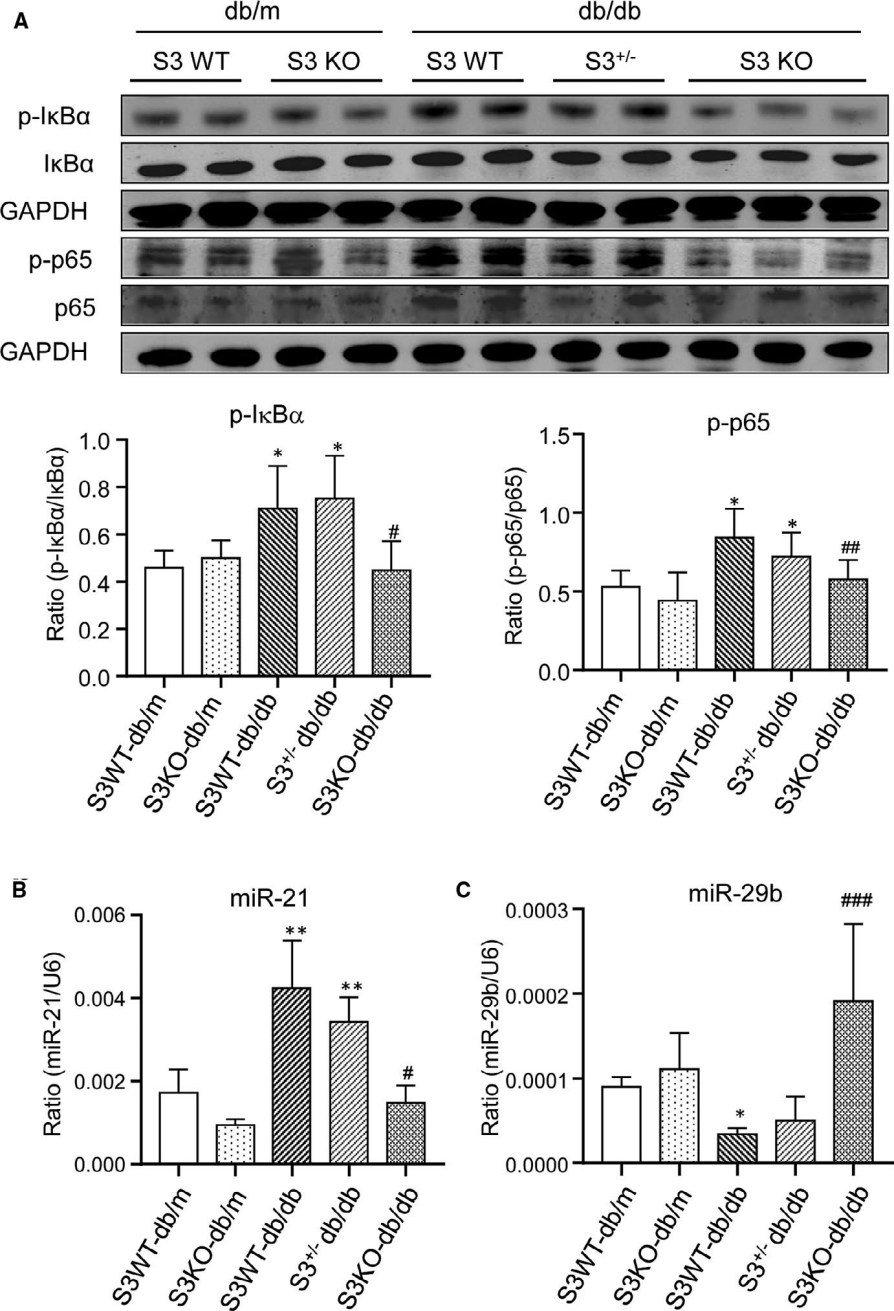
Smad3 deficiency inactivates cardiac NF‐kB signalling and suppresses myocardial miR‐21 while up‐regulating miR‐29b in db/db mice. A, Western blot analysis of p‐IκBα and p‐p65. B, C, Real‐time PCR analysis of miR‐21 and miR‐29b. Values are expressed as mean ± SE for group of eight mice. *P 05 and **P 01 compared with Smad3(S3) WT‐db/m group; ^#^ *P* 05, ^##^ *P* 01 and ^###^ *P* 001 compared with Smad3 (S3) WT‐db/db and Smad3^+/−^db/db groups

Previously, we reported that Smad7 is capable of inducing IκBα, an inhibitor of NF‐κB, to inhibit renal inflammation.^11^, ^12^, ^13^ We also report that mice null for Smad3 are resistant to angiotensin II‐induced renal inflammation by inhibiting the Smad ubiquitination regulatory factor 2 (Smurf2)‐mediated degradation of renal Smad7.^20^ We then tested a hypothesis that the protection of Smad3 KO‐db/db mice from cardiac inflammation may be associated with the inactivation of NF‐κB signalling by blocking Smurf2‐mediated ubiquitin degradation of cardiac Smad7. As expected, Western blot analysis revealed that compared to the Smad3 WT‐db/db and Smad3^+/−^ db/db mice where a dramatic up‐regulation of cardiac Smurf2 was related to a loss of Smad7 and activation of NF‐κB signalling, and Smad3 KO‐db/db mice showed a significant down‐regulation of cardiac Smurf2 but up‐regulation of cardiac Smad7 (Figure 5E‐H), thereby blocking a marked phosphorylation of IκBα and NF‐κB/p65 (Figure 6A).

We have also previously detected that Smad3 mediates cardiac and renal fibrosis and inflammation in hypertensive heart disease and diabetic nephropathy by up‐regulating miR‐21 while down‐regulating miR‐29b.^21^, ^22^, ^23^ Similarly, increased cardiac TGF‐β/Smad3 signalling was correlated with up‐regulation of miR‐21 but down‐regulation of miR‐29b in Smad3 WT‐db/db and mice, which was reversed in Smad3 KO‐db/db mice (Figure 6B,C).

## 4 DISCUSSION

In the current study, we demonstrated that Smad3 plays a pathogenic role in DCM. This was demonstrated by the findings that db/db mice lacking Smad3 were protected against cardiac dysfunction including the fall in LVEF and LVFS and an increase in LV mass, thereby preventing progressive cardiac inflammation and fibrosis.

It is well established that TGF‐β is a master regulatory in tissue fibrosis.^24^, ^25^ TGF‐β1 mediates fibrosis via its downstream Smad3, but not Smad2, signalling.^26^ In the heart, fibroblast‐specific deletion of Smad3, not Smad2, can markedly inhibit cardiac fibrosis in the infarcted myocardium.^7^, ^8^, ^9^, ^10^ Moreover, Smad3 deficiency is also reported to inhibit angiotensin II‐induced hypertensive cardiovascular diseases.^6^, ^16^ In diabetes, mice lacking Smad3 are protected against both renal and cardiac fibrosis in streptozotocin (STZ)‐induced type 1 diabetes and in obese diabetic mice.^10^, ^27^, ^28^ In the present study, we found that a complete loss of Smad3 in db/db mice was required to prevent the development of DCM as Smad3 haploinsufficiency (Smad3^+/−^ db/db) mice, like Smad3 WT‐db/db mice, showed no protective effect on cardiac dysfunction and progressive myocardial inflammation and fibrosis. These observations were consistent with the previous report that Smad3^−/−^ db/db, but not Smad3^+/−^ db/db mice and Smad3^+/+^db/db mice, are protected against the development of type‐2 diabetic phenotypes and diabetic nephropathy.^17^, ^19^ These cardiorenal protective effects may be attributed largely to the prevention of db/db mice from the development of diabetic phenotypes such as hyperglycaemia, hyperlipidaemia, hypertension, glucose intolerance, insulin resistance and obesity in Smad3 KO‐db/db, but not in Smad3^+/−^db/db and Smad3 WT‐db/db mice as seen in this and other studies.^10^, ^19^ Thus, results obtained from this and other studies reveal an essential role for Smad3 in the pathogenesis of type 2 diabetes and diabetic cardiorenal complications.

**FIGURE 7:**
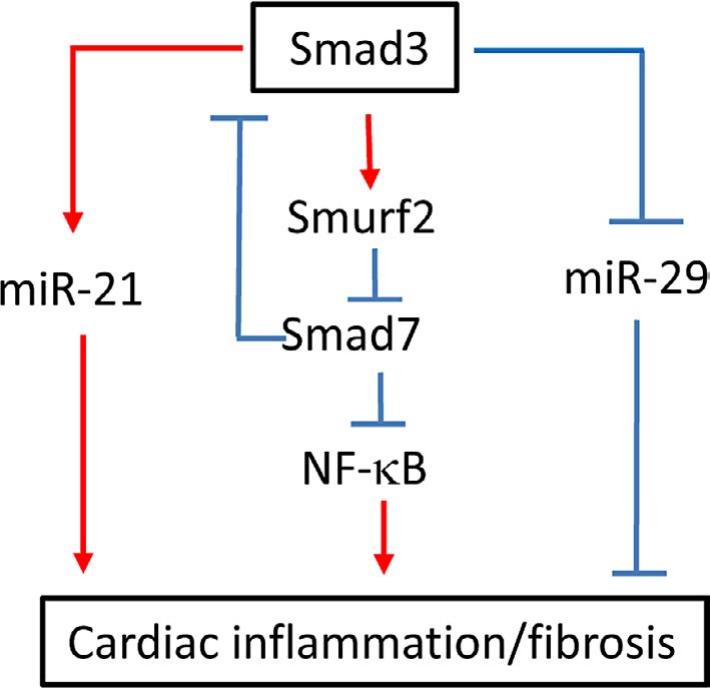
Diagram of Smad3‐mediated cardiac inflammation and fibrosis. Smad3 mediates cardiac fibrosis by up‐regulating miR‐21 while down‐regulating miR‐29. Smad3 also induces degradation of an inhibitory Smad7 by up‐regulating an E3‐ligase Smurf2, which results in cardiac inflammation and fibrosis by promoting activation of NF‐κB and Smad3 signalling

As illustrated in Figure 7, Smad3 may mediate cardiac inflammation and fibrosis via two major mechanisms. First, prevention of cardiac Smad7 from Smurf2‐dependent ubiquitin proteasomal degradation may be a mechanism though which deletion of Smad3 from db/db mice was protected against cardiac inflammation and fibrosis. We have previously shown that Smad7 is a unique inhibitor for both TGF‐β/Smad and NF‐B signalling.^11^, ^12^, ^13^ It is known that Smad7 binds to Smurf2 to form an E3 ubiquitin ligase that targets the TGF‐β receptor for degradation.^29^ In the process of tissue fibrosis, overreactive Smad3 is associated with a loss of Smad7 protein due to up‐regulation of Smurf2.^11^, ^12^, ^30^ Smad7 can also function to induce expression of IκBα, an inhibitor of NF‐κB, to negatively regulate NF‐κB‐driven inflammatory response.^13^ Furthermore, Smad7 promoter contains a putative NF‐κB regulatory site.^31^ Thus, once Smad7 is degraded, both TGF‐β/Smad and NF‐κB signalling are overreactive. Therefore, disrupted Smad7 increases but overexpression of myocardial Smad7 inhibits angiotensin II‐induced cardiac fibrosis and inflammation.^11^, ^12^ Consistent with these findings, we also discovered that Smad3‐null db/db mice were resistant to development of cardiac inflammation by suppressing expression of IL‐1β, TNF‐α and macrophage infiltration.^6^ This was associated with inactivating NF‐κB/p65 signalling by up‐regulating cardiac Smad7.

Rebalancing the Smad3‐dependent anti‐ and pro‐fibrogenetic miRNAs could be another mechanism through which deletion of Smad3 protects against myocardial fibrosis in Smad3 KO‐db/db mice. It has also been shown that TGF‐β mediates tissue fibrosis via Smad3‐dependent miRNAs such as miR‐21 and miR‐29.^21^, ^22^, ^23^, ^24^, ^25^, ^32^, ^33^ It is now well established that Smad3 mediates cardiorenal fibrosis under diabetic and hypertensive conditions by down‐regulating miR‐29 while up‐regulating miR‐21.^21^, ^22^, ^23^, ^24^, ^25^, ^32^, ^33^ Therefore, overexpression of miR‐29 or inhibition of miR‐21 can attenuate cardiorenal fibrosis and inflammation under hypertensive and diabetic conditions. In line with these findings, the current study also found that prevention of cardiac inflammation and fibrosis in db/db mice lacking Smad3 was associated with an increase in cardiac miR‐29b while inhibiting miR‐21expression. It has been reported that miR‐21 can target Smad7 to induce renal fibrosis.^22^ It is also possible that up‐regulation of miR‐21 may inhibit cardiac Smad7 expression, resulting in Smad3‐mediated cardiac fibrosis and inflammation as seen in Smad3 WT‐db/db and Smad^+/−^db/db mice. Furthermore, as Smad3 can induce pro‐inflammatory cytokine MCP‐1 via binding directly to its promoter or via Smad3‐dependent LRNA9884 mechanism^34^; thus, loss of Smad3 may also contribute to MCP‐1‐dependent macrophage inflammation in the heart of diabetic db/db mice.

## CONFLICT OF INTEREST

The authors declare that there are no conflicts of interest.

## AUTHOR CONTRIBUTIONS

**Li Dong:** Data curation (equal); Formal analysis (equal); Investigation (equal); Methodology (equal); Visualization (equal). **Jian‐chun Li:** Data curation (equal); Methodology (equal); Writing‐original draft (equal). **Zhong‐Jing Hu:** Investigation (equal); Methodology (equal). **Xiaoru Huang:** Methodology (equal). **li wang:** Methodology (equal). **Hong‐lian Wang:** Data curation (equal); Investigation (equal); Methodology (equal). **Ronald CW Ma:** Data curation (equal); Investigation (equal); Methodology (equal); Supervision (equal). **Hui‐yao Lan:** Conceptualization (equal); Funding acquisition (equal); Investigation (equal); Project administration (equal); Resources (equal); Supervision (equal); Validation (equal); Writing‐review & editing (equal). **Si‐Jin Yang:** Conceptualization (equal); Funding acquisition (equal); Investigation (equal); Project administration (equal); Resources (equal); Supervision (equal); Validation (equal); Writing‐review & editing (equal).

## Supporting information

Fig S1

## Data Availability

The data that support the findings of this study are available from the corresponding author upon reasonable request.
