# Supplementary material for: Deletion of Smad3 protects against diabetic myocardiopathy in db/db mice
Source: J Cell Mol Med. 2021 Mar 17;25(10):4860–9. doi: 10.1111/jcmm.16464 (PMC8107104; doi:10.1111/jcmm.16464)
Supplement: Supplementary file 1 — Fig S1 [file JCMM-25-4860-s001.pdf]

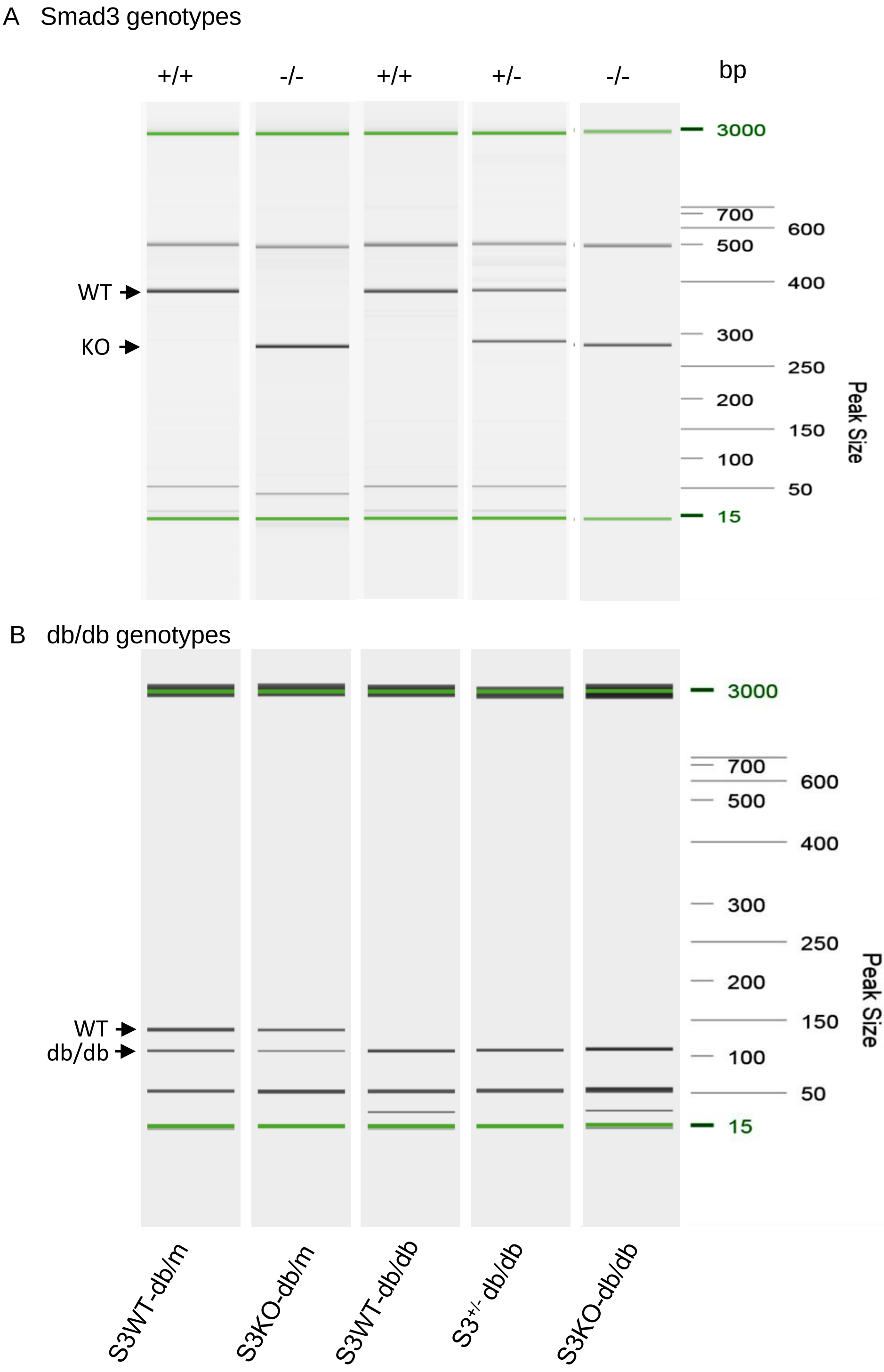

Supplementary Figure 1. Genotypic determination of Smad3 KO from db/db mice. PCR with individual primers specific to Smad3 or db/db shows that Smad3 WT (431bp) and Smad3 KO (248bp) in db/m (WT 135bp) and db/db (108bp) mice are clearly identified and 5 mouse genotypes are determined.
